# Intracellular Delivery of Therapeutic Protein via Ultrathin Layered Double Hydroxide Nanosheets

**DOI:** 10.3390/pharmaceutics16030422

**Published:** 2024-03-19

**Authors:** He Zhang, Anle Ge, Yulin Wang, Boran Xia, Xichu Wang, Zhonghui Zheng, Changsheng Wei, Bo Ma, Lin Zhu, Rose Amal, Sung Lai Jimmy Yun, Zi Gu

**Affiliations:** 1 School of Chemical Engineering, University of New South Wales, Sydney, NSW 2052, Australia; 2 Single-Cell Center, CAS Key Laboratory of Biofuels, Shandong Key Laboratory of Energy Genetics, Shandong Energy Institute, Qingdao Institute of Bioenergy and Bioprocess Technology, Chinese Academy of Sciences, Qingdao 266101, China; 3 Australian Carbon Materials Centre (A-CMC), School of Chemical Engineering, University of New South Wales, Sydney, NSW 2052, Australia; 4 Shandong Xinhua Pharmaceutical Co., Ltd., Zibo 255086, China; 5 College of Chemical and Pharmaceutical Engineering, Hebei University of Science and Technology, Shijiazhuang 050018, China; 6 School of Mechanical Engineering, Qingdao University of Technology, Qingdao 266520, China

**Keywords:** nanomedicine, protein delivery, two-dimensional (2D) nanoparticle, layered double hydroxide, cancer therapy

## Abstract

The therapeutic application of biofunctional proteins relies on their intracellular delivery, which is hindered by poor cellular uptake and transport from endosomes to cytoplasm. Herein, we constructed a two-dimensional (2D) ultrathin layered double hydroxide (LDH) nanosheet for the intracellular delivery of a cell-impermeable protein, gelonin, towards efficient and specific cancer treatment. The LDH nanosheet was synthesized via a facile method without using exfoliation agents and showed a high loading capacity of proteins (up to 182%). Using 2D and 3D 4T1 breast cancer cell models, LDH–gelonin demonstrated significantly higher cellular uptake efficiency, favorable endosome escape ability, and deep tumor penetration performance, leading to a higher anticancer efficiency, in comparison to free gelonin. This work provides a promising strategy and a generalized nanoplatform to efficiently deliver biofunctional proteins to unlock their therapeutic potential for cancer treatment.

## 1. Introduction

Bioactive proteins perform an imperative function in living organisms, and their ability to be delivered into cells for purposes of diagnostics and therapeutics is an attractive prospect. Protein therapy differs from gene therapy or small molecular drugs in that it targets the disease rather than randomly integrating genetic material, resulting in highly efficient therapeutic outcomes with minimal side effects [1,2]. There is growing evidence that protein compounds exert their biological effects by inhibiting intracellular biological processes [3]. Nevertheless, the development of an efficient and safe intracellular delivery protein remains a major challenge due to the intrinsic characteristics of most proteins as well as the biological cell membrane barrier.

In most cases, native proteins are serum-instable, prone to degradation, and rendered inactive after administration [4]. Additionally, certain protein toxins cannot pass through the cell membrane due to the lack of type II domains, such as class I ribosome-inactivating proteins (RIPs) [5]. A number of potential anticancer drugs have been developed from RIPs, which inhibit protein synthesis to an incredibly high degree [6]. Gelonin is a typical class I RIP consisting of a glycoprotein with a single chain around 30 kDa in molecular weight, produced from the plant *Gelinium multiforum* [7]. Gelonin, however, has not been clinically tested as a therapeutic agent because of its poor intracellular delivery ability and low endocytotic activity, which necessitates an efficient delivery strategy to unlock its therapeutic potential.

The most extensively investigated method of delivering proteins to cells in recent decades is the fusion of the protein with protein transduction domains or cell-penetrating peptides [8,9]. By using either method, the fused protein can be delivered into the cells through endocytosis. These cargo proteins would normally be entrapped within endosomes and would therefore not be able to escape into the cytosol, yielding a low intracellular delivery efficiency [10,11]. As a result, the development of a suitable protein delivery system should not only take into account the preservation of the therapeutic activity of protein cargo in the serum, but also the delivery of the protein directly into the cytoplasm. The use of nanoparticles for protein delivery has gained attention in recent years owing to numerous distinct properties at nanoscale that make them suitable options for delivering proteins intracellularly [12]. Liposomes, lipids, and polymeric and organic nanoparticles have been commonly used for protein delivery, enhancing the stability, bioactivity, and therapeutic efficacy of the cargo protein [12].

**Figure 1:**
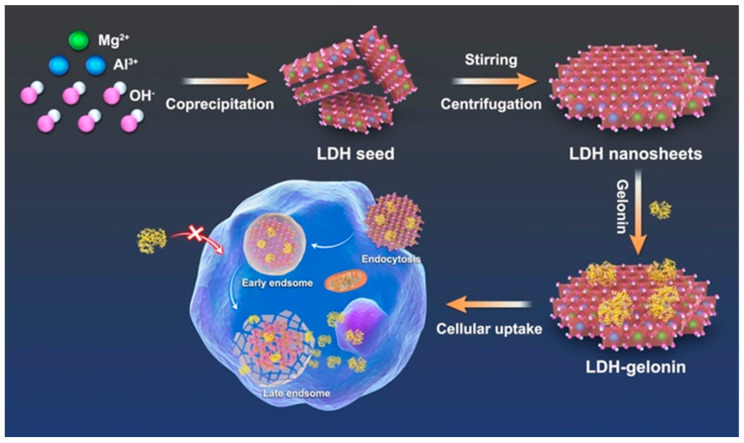
Schematic graphic of the synthesis and intracellular protein delivery of LDH nanosheets (mass ratio of LDH to gelonin 5:6).

Layered double hydroxide (LDH) nanoparticles and nanosheets are a large family of two-dimensional (2D) lamellar clay nanoparticles that have shown extensive potential as an effective drug delivery system [13,14]. LDH is capable of degradation and releasing loaded drugs in a sustained manner in an acidic physiological environment, making it highly biosafe and effective in comparison with other delivery systems [15,16]. The buffering effect of LDH nanoparticles may serve to prevent the denaturation and premature degradation of cargos in the biological environment, which is appropriate for protein delivery [17]. Moreover, the presence of a positive charge in the LDH layer makes it very effective at permeating cell membranes, which allows it to enhance intracellular drug delivery. LDH nanoparticles exhibit the ability to escape endosomes, thereby preventing bioactive agents from degradation within acidic endosomal or lysosomal environments and facilitating their release into the cytoplasm [18,19]. Based on our previous research, proteins such as bovine serum albumin (BSA) could be loaded on LDH nanoparticles. However, the loading capacity is restricted by the lamellar structure of traditional LDH nanoparticles. Herein, in this work, an ultrathin, fewer-layer LDH nanosheet was synthesized for the intracellular delivery of gelonin to efficiently kill cancer cells. The LDH nanosheet was prepared using a facile bottom-up method without using any exfoliation agents, and was aged at room temperature, exhibiting a relatively small lateral size of 30 nm and a thickness of 2.5 nm (Figure 1). BSA was used as a model protein to demonstrate the superior protein adsorption ability of the LDH nanosheet, and then gelonin was loaded on the nanosheet to evaluate binding affinity, loading capacity, and pH-responsive protein release. The therapeutic potential of LDH–gelonin nanohybrids was demonstrated in 2D 4T1 breast cancer cell cultures and 3D 4T1 spheroids in terms of cytotoxicity, apoptosis, and tissue penetration. The LDH nanosheets were also revealed to facilitate the cellular uptake and endosomal escape of gelonin for cytoplasm delivery. This proof-of-principle work demonstrated a promising therapeutic protein delivery system using ultrathin LDH nanosheets towards efficient, specific cancer therapy.

## 2. Methods and Materials

### 2.1. Materials and Reagents

MgCl_2_·6H_2_O (≥99.0%), AlCl_3_·6H_2_O (99.99%), NaOH (99.9%), and albumin from bovine serum (BSA) were purchased from Aladdin (Bay City, MI, USA). Gelonin was bought from Enzo^®^ (Farmingdale, NY, USA). The Alexa Fluor™ 488 protein labeling kit and penicillin/streptomycin were bought from ThermoFisher SCIENTIFIC (Waltham, MA, USA). Phosphate buffer solution (PBS) and Dulbecco’s Modified Eagle’s Medium (DMEM, high glucose) were purchased from Sigma-Aldrich (St. Louis, MO, USA). CellTiter-Glo Luminescent Cell Viability Assay was purchased from Promega (Madison, WI, USA). The Annexin V-FTIC apoptosis detection kit was bought from YEASEN company (Shanghai, China). DAPI staining solution was purchased from Abcam (Boston, MA, USA). LysoTracker Deep red was bought from Thermo Fisher. The mammalian protein extraction kit was purchased from CWBIO company (Taizhou, China). 4T1 cells were bought from Procell life science & technology co, Ltd. (Wuhan, China). The CellTiter-Glo^®^ 3D cell viability assay kit was brought from Promega. A scaffold for 3D cell culture was purchased from Tantti (Taoyuan City, China). Milli-Q water was used in the experiments.

### 2.2. Synthesis of LDH Nanosheets

LDH nanosheets were prepared using a coprecipitation method at room temperature. Specifically, 10 mL of solution A containing 3 mmol MgCl_2_•6H_2_O and 1 mmol AlCl_3_•6H_2_O was rapidly added into 40 mL of solution B containing 6 mmol NaOH while stirring vigorously for 10 min. Following centrifugation for 5 min at 4000× *g* rpm, the resulting slurry was resuspended in 40 mL water and stirred for 16 h at 600 rpm under room temperature. The LDH nanosheet was collected by means of centrifugation at 4000× *g* rpm to remove aggregated particles, and then redispersed in 40 mL water. The concentration of LDH nanosheet suspension was measured to be 3.69 mg/mL, and the yield was 31%.

### 2.3. Synthesis of LDH–Protein Nanohybrids

BSA-modified LDH particles were synthesized according to a previous report [20]. Briefly, 1 mL LDH (2 mg/mL) suspension was added into 4 mL BSA solution (ranging from 0.125 to 10 mg/mL) drop by drop under vigorous stirring for 2 h at room temperature. After centrifugation at 10,000× *g* rpm for 10 min, the supernatant was collected for UV-vis testing to determine the BSA loading capacity (LC) and loading efficiency (LE).

The LC and LE of protein loading on nanoparticles were calculated by using the following equations:

LC (%) = (total protein mass − protein mass in supernatant)/nanoparticle mass × 100%

LE (%) = (total protein mass − protein mass in supernatant)/total drug mass × 100%

The gelonin was labeled with Alexa488 using an Alexa Fluor™ 488 Protein Labeling Kit (gelonin-488) according to the product protocol. The gelonin- or Alexa488-gelonin-modified LDH (LDH–gelonin, LDH–gelonin-488) were prepared following the procedure described above.

### 2.4. Characterizations

Transmission electron microscopy images were obtained on a JEOL JEM-F200 electron microscope operated at an accelerating voltage of 200 kV. X-ray diffraction (XRD) patterns were recorded on an Empyrean Thin-Film XRD (PANalytical Empyrean) using Cu Ka source at a scanning rate of 0.01°/min from 2θ = 5° to 55°. Dynamic light scattering (DLS) and zeta potential measurement were conducted on a Nanosizer Nano ZS instrument (Malvern, Malvern, UK). Atomic force microscopy (AFM) was performed on a NanoScope IIIa from Veeco Instruments (Plainview, NY, USA). UV-vis absorption spectra were obtained in the range of 200 to 700 nm using a Shimadzu U-3000 spectrophotometer (Shimadzu, Kyoto, Japan), and the slit width was 1.0 nm.

### 2.5. Colloid Stability Testing

Five ml of LDH–gelonin suspension were mixed with 5 mL of water, saline, PBS, and DMEM under stirring for 15 min, and the hydrodynamic particle size was then measured via DLS.

### 2.6. Sodium Dodecyl Sulphate–Polyacrylamide Gel Electrophoresis (SDS-PAGE)

For the determination of the binding affinity of the protein to LDH nanosheets, the SDS-Page gels were carried out on 180 V for 60 min. In detail, different mass ratios of gelonin and LDH nanosheets (ranging from 1:0.1 to 1:4) were mixed via vortex for 30 s, and then kept under shaken bed for 30 min at room temperature. The prepared samples were then mixed with loading buffer X1 with a volume ratio of 1:1 and incubated at 100 °C for 10 min denaturation. After centrifugation at 12,000× *g* rpm for 5 min, the denatured samples were loaded on the gel well and subjected to electrophoresis at 180 V for 60 min.

In order to evaluate the release capability of protein from LDH nanosheets under the acidic pH level to mimic the environment of early endosomes [21], firstly, gelonin and LDH nanosheets were mixed with a mass ratio of 1:1. Upon spinning down the mixture, the pellets were suspended in a buffer containing pH values of 6.0 and 7.4 at 37 °C in a thermal mixer for 24 h. Next, various samples containing gelonin in pH 6.3 buffer (2 μg/μL), LDH–gelonin (1:1) in pH 7.4, LDH–gelonin (1:1) in pH 6.0, and LDH nanosheets in pH 6.0, were subjected to gel electrophoresis as described above.

### 2.7. In Vitro Antitumor Efficiency Evaluation

The 4T1 cells were seeded in 96-well black microplates at a density of 10^4^ cells/well for overnight attachment. Upon reaching a confluency of 50%, 100 μL of fresh media containing PBS, LDH nanosheets, gelonin, and LDH–gelonin were added to each well at various concentrations of gelonin (0.5, 1, 2, 3, 6, and 8 μg/mL) and the corresponding LDH concentrations (0.4, 0.8, 1.6, 2.4, 4.8, and 6.4 μg/mL). The cell viability was determined after a 24 h incubation using the CellTiter-Glo Luminescent Cell Viability Assay and was calculated in comparison to the PBS control.

To visualize the live and dead cells, 4T1 cells were seeded in a 6-well plate at a density of 5 × 10^5^ cells/well. The culture medium was replaced with medium containing PBS, gelonin (6 µg/mL), LDH (5 μg/mL), and LDH–gelonin (11 µg/mL). After a 24 h incubation, the cells were stained with a calcein acetoxymethyl ester solution (50 µL, 20 mM) and a PI solution (50 µL, 20 mM) to visualize live cells (green; λex = 490 nm, λem = 515 nm) and dead cells (red; λex = 535 nm, λem = 615 nm), respectively. Following another 15 min incubation, the stain solutions were removed, and the wells were rinsed twice with PBS. Afterward, the cells were observed under a fluorescence microscope (Olympus, Tokyo, Japan, CKX53).

Apoptosis analysis via flow cytometry: The 4T1 cells were seeded in a 6-well plate at a density of 5 × 104 cells/well for overnight attachment to achieve 50% confluence, and then the culture medium was replaced with PBS, gelonin (6 µg/mL), LDH–gelonin (11 µg/mL), and LDH (5 µg/mL). After a 24 h incubation, the 4T1 cells were collected by using trypsin without EDTA via centrifugation at 300× *g* at 4 °C for 5 min. Immediately, the collected cells were washed twice with pre-cooled PBS and then resuspended in 100 µL 1 × binding buffer. Subsequently, 5 µL Annexin V-FITC and 10 µL PI staining solution were added and mixed gently at room temperature for 10–15 min. After supplementing with another 400 µL 1 × binding buffer into each sample, all samples were placed on ice for measurement using flow cytometry (BD FACSAria™ II, Franklin Lakes, NJ, USA).

### 2.8. In Vitro Cellular Localization of LDH–Gelonin Nanohybrids

The 4T1 cells were seeded in a 6-well plate at a density of 5 × 10^4^ cells/well. At approximately 50% confluency, the culture medium was replaced with media containing PBS, gelonin-488 (6 µg/mL), LDH (5 µg/mL), and LDH–gelonin-488 (11 µg/mL). The cells were washed with PBS three times following a 4 h and 24 h incubation. After that, the lysotracker deep red was added in accordance with the product protocol, and the cells were incubated for 1 h before being washed 3 times in PBS. DAPI was then added, and the cells were stained for another 5 min. The cells were observed under a confocal microscope (Olympus FV1000).

### 2.9. Western Blot Analysis

The 4T1 cells were seeded in a 6-well plate at a density of 5 × 10^4^ cells/well. At approximately 50% confluency, the culture medium was replaced with media containing PBS, LDH, gelonin (6 µg/mL), LDH–gelonin (5.5 µg/mL), and LDH–gelonin (11 µg/mL). After 24 h incubation, the cells were harvested, washed, and then suspended in 200 µL lysis buffer. Protein concentration was quantified using a BCA Protein Assay (BioRad, Hercules, CA, USA). An equal amount of proteins was loaded onto 12% (*w*/*v*) SDS polyacrylamide gel electrophoresis (SDS-PAGE) and then electrically transferred to a polyvinylidene difluoride membrane (Hybond-P, Amersham Biosciences, Piscataway, NJ, USA). The membranes were blocked with tris-buffered saline + 0.1% Tween 20 (TBST) + 5% (*w*/*v*) skim milk, and then incubated overnight at a 1:1000 dilution of primary antibodies (anti-Bax (CST, rabbit), anti-Bcl-2 (CST, rabbit), and anti- GAPDH (CST, rabbit)). Thereafter, the membranes were washed in TBST, incubated with horseradish peroxidase (HRP)-conjugated anti-rabbit IgG (CST, rabbit) for 1 h, and washed again with TBST. The positive bands were visualized using the Amersham ECL Plus Western blotting detection reagents (GE Health care, Piscataway, NJ, USA).

### 2.10. Evaluation of the Tissue Penetration Ability of LDH–Gelonin in 3D Tumor Spheroids

Three-dimensional spheroids were prepared through the incubation of 4T1 cells (40 µL) at a density of 5 × 10^6^/mL in a 48-well plate containing scaffolds (Tantti^®^ SpherTantrix) for 45–60 min. After that, 260 µL of fresh cell culture medium was added to each well and incubated for an additional 3 days. Next, the 4T1 cell spheroids were incubated with LDH–gelonin-488 (11 µg/mL) nanoparticles for 12 h, and then washed twice with PBS. The fluorescence images with various depths (z-stacking) were obtained using confocal microscopy (Olympus FV1000).

### 2.11. Antitumor Evaluation

The antitumor efficacy of LDH–gelonin on the 3D 4T1 spheroids was evaluated using a Celltiter Glo Assay kit (Promega). The spheroids were incubated with different concentrations of PBS, LDH, gelonin, and LDH–gelonin (normalized to gelonin, 1, 2, 3, 6, and 8 μg/mL) in a 96-well black plate containing 3D scaffolds. Following a 48 h incubation, the culture medium was replaced with 200 mL PBS and then representative brightfield images of the 4T1 spheroids were obtained using an Olympus CKX53 inverted microscope equipped with an UPlanFL N 10x/0.13na objective. The cell viability of the 3D spheroids was measured using the CellTiter-Glo^®^ 3D cell viability assay kit.

## 3. Results and Discussion

### 3.1. Fabrication and Characterization of LDH Nanosheets and LDH–Protein Nanohybrids

**Figure 2:**
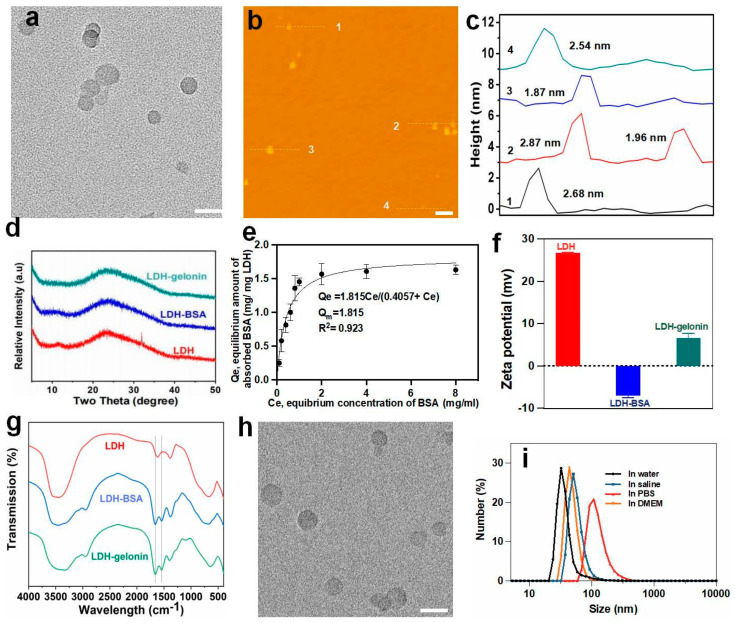
Physicochemical structures of LDH nanosheets, LDH-BSA, and LDH–gelonin, and protein binding capacity of LDH nanosheets. (**a**) TEM image of LDH nanosheets. Scale bar = 50 nm. (**b**) and (**c**) AFM image and corresponding thickness of LDH nanosheets, respectively. Scale bar = 100 nm. (**d**) XRD patterns of LDH nanosheets, LDH-BSA, and LDH–gelonin. (**e**) Adsorption isotherm of BSA on LDH nanosheets fitted in Langmuir model with R^2^ being 0.923 and the maximum monolayer adsorption capacity (Q_m_) being 1.82 mg (BSA) per mg LDH. The data (n = 2) are expressed as mean ± SD. (**f**) Zeta potential (n = 3) and (**g**) FTIR spectra of LDH nanosheets, LDH-BSA, and LDH–gelonin. (**h**) TEM images of LDH–gelonin. Scale bar = 50 nm. (**i**) Size distribution of LDH–gelonin in water, saline, DMEM, or PBS.

As shown in Figure 1, LDH nanosheets were fabricated via a facile co-precipitation method at room temperature. Specifically, after mixing metal salts and alkaline solutions, the LDH seed nanoparticles were formed and showed a wide hydrodynamic size distribution of about 497 nm in an average size and a PDI of 0.494 (Figure S1, Supporting Information). After continuous stirring for another 16 h, the final LDH nanosheet suspension was obtained, showing an average hydrodynamic particle size of 33 nm and a PDI of 0.245. Transmission electron microscopy (TEM) images of LDH nanosheets demonstrated an orbicular morphology and an average lateral dimension of 29 nm (Figure 2a), which is consistent with DLS measurement results. An atomic force microscope (AFM) was used to measure the thickness of the LDH nanosheets (Figure 2b,c). The results demonstrated that LDH was about 2.5 nm in thickness, suggesting a few-layered nanosheet structure containing only three to four hydroxide layers. The X-ray diffraction pattern of the LDH nanosheets showed a weak (003) diffraction peak at 11.57°, from which the nanosheet thickness was calculated to be 2.81 nm, in accord with the AFM results.

Considering that the 2D nanosheet structure could provide more cargo loading sites, we then used BSA as a model protein to evaluate the maximum protein loading capacity of the LDH nanosheets. BSA was loaded on the LDH nanosheets via a dropwise addition method that we previously reported [14], and the mass of BSA bound to the nanosheets was calculated. The adsorption data were well fitted in the Langmuir model (R^2^ = 0.923). The maximum monolayer adsorption capacity (Q_m_) was calculated as 1.82 mg BSA per mg LDH nanosheets, which marks a 2.6-fold increase in comparison to that of the traditional LDH nanoparticles (0.7 mg mg^−1^) (Figure 2e) [20]. The increased Q_m_ value could be attributed to the high specific surface area that is associated with the ultrathin structure (~2.5 nm) and the small lateral diameter (~30 nm) of the LDH nanosheets.

Encouraged by the high protein loading capacity, gelonin, a potential anticancer therapeutic protein, was loaded on LDH nanosheets using the same dropwise addition method to form LDH–gelonin nanohybrids. The zeta potential of LDH–gelonin was measured to be +6.5 ± 1.1 mV, which is remarkably lower than that of LDH nanosheets (+26.7 ± 0.2 mV), indicating the loading of gelonin on LDHs via electrophoresis interaction (Figure 2f; Table S2, Supporting Information). The FTIR spectra of LDH–gelonin showed apparent absorption peaks at 1660 cm^−1^ and 1545 cm^−1^ that were not observed in the FTIR spectrum of the unconjugated LDH nanosheets (Figure 2g). The peaks are attributed to stretching vibrations of C=O groups and bending vibrations of N-H in proteins [20,22], further confirming the successful conjugation of gelonin on LDH nanosheets. TEM images of LDH-gelonin demonstrated a similar morphology to unconjugated LDH nanosheets (Figure 2h). The particle size of LDH–gelonin was measured to be ca. 31 nm from TEM images, which is close to that of the LDH nanosheets. The XRD patterns of LDH–gelonin conjugates appeared similar to that of the LDH nanosheets but showed even weaker and broader (003) diffraction peaks (Figure 2d), from which the nanosheet thickness of LDH–gelonin was measured to be 2.95 nm. Furthermore, LDH–gelonin exhibited desirable colloidal stability in various biological environment-relevant solutions (e.g., saline, PBS, and cell culture medium), showing an average size of 44–83 nm and a PDI of 0.19–0.24 (Figure 2i).

### 3.2. Binding Affinity and Protein Release Evaluation

To determine the binding affinity and loading capacity of gelonin on LDH nanosheets, the binding ability of LDH–gelonin was examined via an SDS-PAGE gel retardation assay. Gelonin was mixed with LDH nanosheets at different mass ratios ranging from 1:0.1 to 1:4, and the hybrids were then subjected to SDS-PAGE, using LDH nanosheets and free gelonin as controls. As shown in Figure S2a, free gelonin migrated and exhibited a bright band in the lane, while no band appeared in the lane of LDH nanosheets, indicating that the band around 30 kDa was attributed to gelonin itself. When the mass ratio of gelonin to LDH nanosheet increased from 1:0.1 to 1:1, the bands of free gelonin became weaker, indicating the increased amount of LDH-bound gelonin. When the mass ratio of gelonin to LDH nanosheet increased to 1:2, no obvious gelonin bands were observed. The results suggest a saturation adsorption of gelonin on the LDH nanosheet at a mass ratio between 1:1 to 1:2. To further quantify the gelonin loading capacity, gelonin and LDH nanoparticles were mixed in a mass ratio of 2.1:1 by dropwisely adding 40 μL of LDH (1 mg/mL) into 30 μL of gelonin (2.8 mg/mL) followed by 4 h shaking. After that, the supernatant was collected via high-speed centrifugation, and gelonin concentration was measured via UV-vis spectrophotometry. The loading capacity of gelonin to LDH nanosheets was determined to be 122% at the mass ratio of gelonin to LDH being 2.1:1, in corresponding to the SDS-PAGE results (Table S1).

To examine gelonin release behavior, LDH–gelonin was dispersed in the buffers of different pH values (pH 7.4 mimicking blood circulation and pH 6.0 mimicking the endosomal environment) for 1 h and then subjected to SDS-PAGE. The gel image showed clear and bright bands in the lanes of free gelonin and LDH–gelonin at pH 6.0 and a much weaker band in the lane of LDH–gelonin at pH 7.4 (Figure S2b). The results suggest the pH-responsive gelonin release of LDH–gelonin, in which gelonin could be released specifically in the endosomal compartments but remain attached on LDH and avoid off-target release in blood circulation.

### 3.3. In Vitro Cytotoxicity and Apoptosis

**Figure 3:**
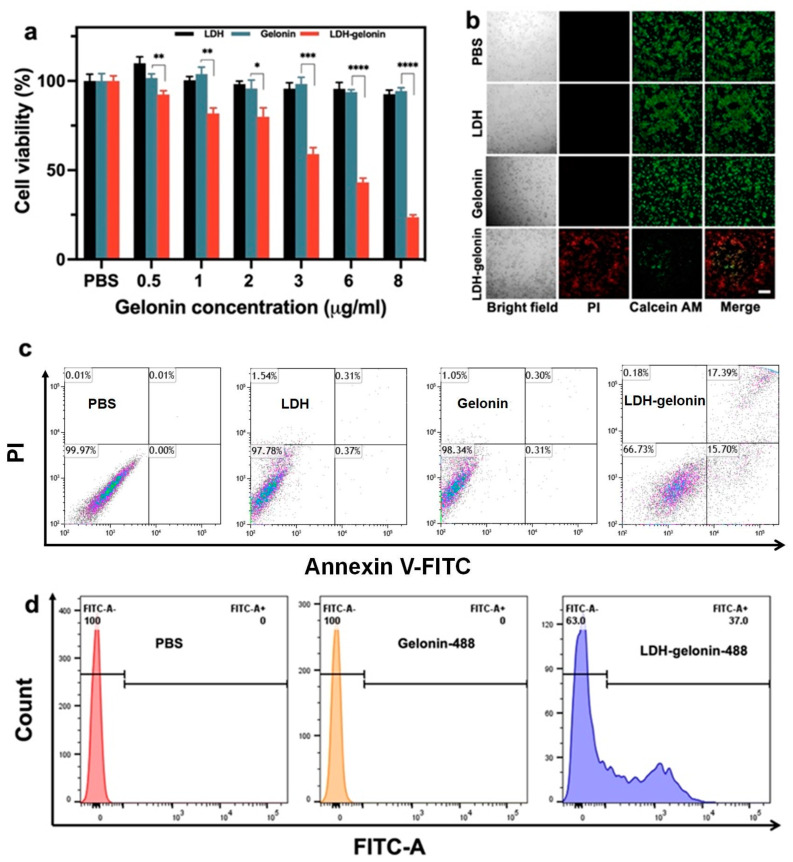
In vitro cell viability studies. (**a**) Cell viability of 4T1 incubated with PBS, LDH, gelonin, and LDH–gelonin with equivalent gelonin concentrations (0.5–8 µg/mL) for 24 h (n = 3). * *p* < 0.05, ** *p* < 0.01, *** *p* < 0.001, **** *p* < 0.0001. (**b**) Fluorescence images of live and dead cells for 24 h incubation with PBS, LDH (5 µg/mL), gelonin (6 µg/mL), and LDH–gelonin (11 µg/mL). Green and red fluorescence indicate live and dead cells via Calcein AM and PI staining (scale bar: 50 µm). (**c**) Apoptosis analysis of 4T1 cells incubated with PBS, LDH (5 µg/mL), gelonin (6 µg/mL), and LDH–gelonin (11 µg/mL) via flow cytometry. (**d**) Cellular uptake evaluation of 4T1 cells after treating with PBS, gelonin (6 µg/mL), and LDH–gelonin (11 µg/mL) for 4 h.

To study the cytotoxicity of LDH–gelonin, the cell viability was assessed via a CellTiter-Glo luminescent cell viability assay by incubating 4T1 breast cancer cells with LDH–gelonin, gelonin, and LDH nanosheets. The values were normalized to the PBS control. The cell viability of the LDH nanosheets at the concentrations of 0.4 to 7 μg/mL was above 92%, showing no significant influence on cell growth. Similarly, free gelonin did not appear to be cytotoxic to 4T1 cells at the concentrations of 0.5 to 8.0 μg/mL, likely due to their very poor membrane permeability. In contrast, LDH–gelonin was significantly cytotoxic toward 4T1 cells in a dose-dependent manner, showing only 23.6 ± 1.4% of live cells at a low concentration of 15 μg/mL after 24 h incubation (Figure 3a). The CD50 value was calculated to be 9.1 μg/mL (LDH–gelonin). Moreover, the distribution of live and dead cells in the treatment groups was visualized via light and fluorescence microscopy by using Calcein-AM (green) and PI (red) dyes. As shown in Figure 3b and Figure S3, there was a strong green fluorescence signal in the groups of PBS, LDH, and gelonin, showing little cytotoxicity. In contrast, LDH–gelonin presented apparent red fluorescence and cells appeared rounded up, indicating strong cancer cell killing ability. The quantitation of the fluorescence intensity of live 4T1 cells revealed that the intensity of 4T1 cells incubated with LDH or gelonin was similar to the PBS group, while the value of the treatment group treated with LDH–gelonin drastically decreased to 39.9% (Figure S4, Supporting Information). BSA was used as a control protein to be adsorbed on LDH nanosheets and showed no cytotoxicity at the concentrations up to 8 µg/mL (Figure S5, Supporting Information). All results showed that gelonin delivered by the LDH nanosheets exhibited significantly enhanced cytotoxicity to cancer cells.

To understand the cell death pathways, the Annexin V-FITC/PI assay via flow cytometry was used to examine the effects of LDH–gelonin on necrosis and apoptosis in 4T1 cells. Annexin V conjugated with FITC is commonly used to identify the presence of phosphatidylserine, a marker of apoptosis that is transported from the interior to the exterior of the plasma membrane in response to pro-apoptotic signals. Cells with broken membranes can be stained with an impermeable dye, PI, to determine necrosis or late apoptosis [23,24]. LDH–gelonin showed a significantly higher level of apoptosis (36.09%) than LDH (0.68%) and gelonin (0.61%) (Figure 3c). Moreover, it was reported previously that gelonin induced apoptotic cell death through regulating Bcl-2 and Bax protein expressions and exhibiting a reduced ratio of Bcl-2/Bax. Therefore, we reason that LDH–gelonin can enhance the reduction in Bcl-2/Bax expression. To validate this, the level of Bcl-2 and Bax protein expression in the 4T1 cells was assessed via Western blot analysis after the treatment with LDH–gelonin and control agents. The results showed that the expression of Bcl-2/Bax was not significantly different among the treatment groups of PBS, LDH, and gelonin, indicating that cell apoptosis was not compromised as a result of these treatments. In contrast, LDH–gelonin (5.5 µg/mL or 11 µg/mL) significantly reduced the ratio of Bcl-2/Bax expression by 60.8% and 21.8%, respectively, in 4T1 cells in comparison with the PBS group (Figure S6, Supporting Information).

### 3.4. Determination of Cellular Uptake Mechanism of LDH–Gelonin Nanohybrids

Poor membrane permeability is a major issue that limits the anticancer application of gelonin. Gelonin delivered by LDH nanosheets is expected to overcome the obstacle and have improved intracellular delivery efficiency. To study the cellular uptake of LDH–gelonin, Alexa-488-linked gelonin was used to fabricate the LDH–gelonin nanohybrids and incubated with 4T1 cells for 4 h, and the percentage of cells internalizing gelonin was analyzed using flow cytometry. The results showed a significant cellular uptake of LDH–gelonin (37%) after 4 h incubation, but no obvious cellular uptake of free gelonin, which indicates a remarkable enhancement of gelonin by the LDH nanosheets (Figure 3d).

**Figure 4:**
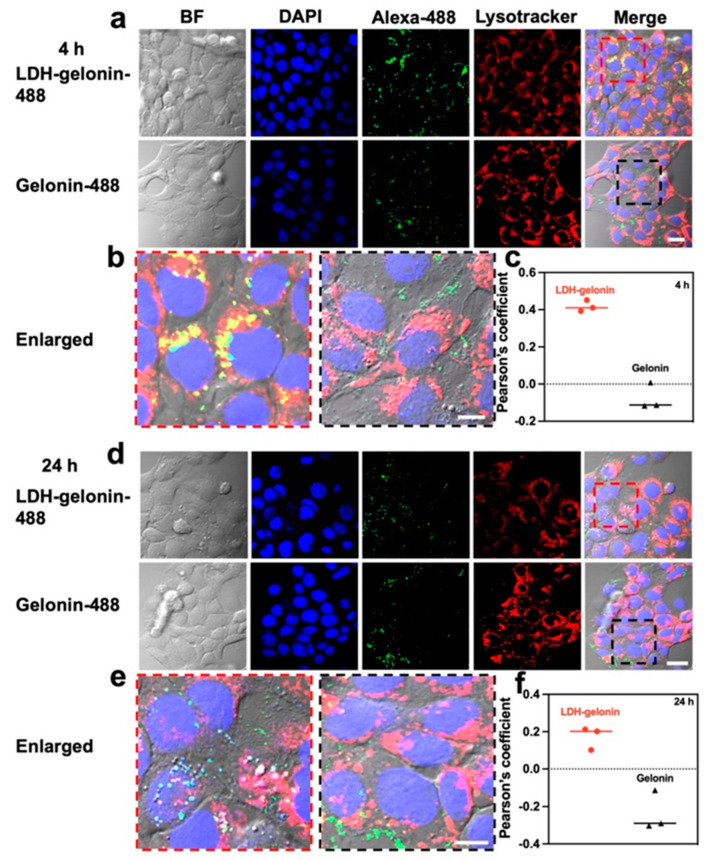
Intracellular colocalization analysis of LDH–gelonin and gelonin samples. (**a**,**b**) and (**d**,**e**) Intracellular colocalization of 4T1 cells incubated with LDH–gelonin-488 and gelonin-488 for 4 h and 24 h, respectively (Blue: DAPI; Green: Alexa-488; Red: Lysotracker. Scale bar = 20 µm). (**c**) and (**f**) Pearson’s coefficient of LDH–gelonin-488 and gelonin-488 after 4 h and 24 h incubation with 4T1 cells, respectively (red dots: LDH–gelonin; black triangles: gelonin).

Due to its susceptibility and fragile nature, gelonin is prone to lose its therapeutic functionality in acidic intracellular organelles [25]. LDH is known to have endosomal escape functionality [19]. By loading gelonin in LDH nanosheets, we expect the LDH carriers to facilitate endosomal escape of gelonin and preserve its therapeutic functionality. To evaluate the cellular uptake pathway and endosomal escape of LDH–gelonin, intracellular trafficking was performed by using fluorescent LDH–gelonin and Lysotracker dye. After 4 h incubation with LDH–gelonin, the cells showed an obvious co-localization of Alexa-488 and Lysotracker (Figure 4a–c), indicating that LDH–gelonin entered lysosomes. After 24 h incubation, the co-localization was dramatically reduced, and a considerable amount of Alexa-488-labelled LDH–gelonin appeared in the cytoplasm (Figure 4d–f), suggesting the release of LDH–gelonin from lysosomes. The endosomal escape of LDH–gelonin was also confirmed by Pearson’s correlation coefficients, which reduced from 0.42 at 4 h to 0.17 at 24 h (Figure 4c,f). In comparison to the LDH–gelonin group, there was a lower level of Alexa-488 signals, and no co-localization was observed in the gelonin group at both 4 h and 24 h, demonstrating the poor intracellular delivery of free gelonin during the whole incubation period.

### 3.5. Tumor Penetration and Antitumor Effect in 3D Tumor Spheroids

**Figure 5:**
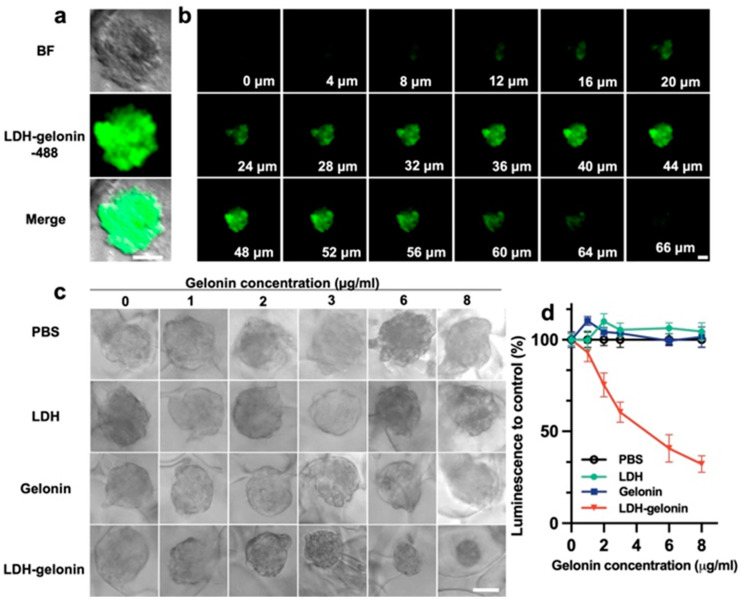
Tumor penetration ability and antitumor activity of LDH–gelonin nanoparticles evaluated on 3D 4T1 spheroids. (**a**) Images of 3D spheroids after 12 h of incubation with LDH–gelonin-488 nanoparticles. (**b**) *z*-axis depth images from the top to the bottom of the treated spheroid. Scanning interval: 4 μm. Scar bar: 50 μm. (**c**) Representative brightfield images of 4T1 tumor spheroids. Images acquired with the Olympus CKX41 inverted microscope with UPlanFL N 10x objective. Scale bar = 50 µm. (**d**) Quantitative analysis of tumor growth inhibition via quantified luminescence intensity obtained from the CellTiter Glo assay (n = 3).

The tumor penetration and antitumor effect of LDH–gelonin were examined in 3D 4T1 tumor spheroids. It has been demonstrated that 3D tumor spheroids provide an effective means of simulating tumor characteristics in order to examine the effects of nanoparticles locally and spatiotemporally on cancer cells [26,27,28]. First of all, 3D spheroids were used to study tumor penetration, which is a major obstacle in nanomedicine delivery. When the 3D spheroids reached around 100 μm, the spheroids were incubated with LDH–gelonin-488. After 12 h incubation, the fluorescence images were recorded using a confocal microscope to examine the penetration of LDH–gelonin-488 in the tumor spheroid. LDH–gelonin showed distinct fluorescence signals along the *z*-axis of the cells (Figure 5a,b), indicating that nanoparticles penetrated deeply into the center of tumor tissues and distributed well throughout the whole spheroid. In comparison, after 12 h of incubation with free gelonin-488, no fluorescence overlapped with 3D spheroids, which suggests that gelonin had a poor ability to penetrate the tumor tissues (Figure S7, Supporting Information).

Next, the antitumor performance of LDH–gelonin nanoparticles was evaluated in 4T1 tumor spheroids via the CellTiter Glo assay. Spheroids with a diameter of 100 μm were incubated with PBS, LDH, gelonin, and LDH–gelonin with an equivalent concentration of gelonin ranging from 0 to 8 μg/mL. Following 48 h incubation, the optical microscopy images of spheroids were obtained and showed remarkably reduced spheroid size in the LDH–gelonin group, while the spheroid size in the LDH and gelonin groups at the corresponding concentrations was not different from that in the PBS control (Figure 5c). Furthermore, the CellTiter Glo assay was used for the quantitative analysis of viable cells in spheroid cultures. The cell viability of the 3D spheroids treated with LDH–gelonin (15 µg/mL) decreased to 32.28%, while the cell viability of LDH and free gelonin groups did not differ significantly from that of the PBS control (Figure 5d). BSA was used as a control protein to be adsorbed on LDH nanosheets and showed little influence on spheroid size and cell viability in spheroid cultures at concentrations up to 8 µg/mL (Figure S8, Supporting Information). Overall, the above findings demonstrated that LDH–gelonin nanoparticles had a superior ability to penetrate and eliminate tumors at a low dosage.

## 4. Conclusions

This work presented the synthesis of LDH nanosheets for efficient intracellular protein delivery for cancer treatment. The 2D LDH nanosheet exhibited a small particle size of 30 nm, an ultrathin structure of 2.5 nm in thickness, and a highly positive charge of +26 mV. Owing to the unique physicochemical properties, the protein loading capacity of the LDH nanosheet was as high as 182%. The cellular studies using 4T1 cancer cell cultures demonstrated the remarkably enhanced cellular uptake efficiency, endosomal escape capacity, and apoptotic cell death of LDH–gelonin in comparison to free gelonin. In 3D tumor spheroids, LDH–gelonin showed excellent tumor penetration and tumor eradication ability at a low dosage. Altogether, this research provides a promising nanoplatform to overcome biological barriers for efficient intracellular protein delivery.

## Data Availability

The data presented in this study are available in this article and Supplementary Material.

## Supplementary Materials

The following supporting information can be downloaded at: https://www.mdpi.com/article/10.3390/pharmaceutics16030422/s1, Table S1: Results of gelonin loading on the LDH nanosheet; Table S2: Summary of hydrodynamic size, PDI, zeta potential and loading capacity of LDH and LDH-protein hybrids; Figure S1: The size distribution of different samples; Figure S2: SDS-PAGE gels showing binding affinity and release study of LDH-gelonin; Figure S3. Fluorescence images of live and dead cells for 24 h incubation with PBS, LDH (5 μg/mL), gelonin (6 μg/mL), and LDH-gelonin (11 μg/mL); Figure S4: Quantification analysis of fluorescence image of live 4T1 cells incubated with PBS, LDH, gelonin or LDH-gelonin; Figure S5: Cell viability of 4T1 incubated with PBS, BSA, LDH, and LDH-BDA with equivalent BSA concentrations (1–8 µg/mL) for 24 h; Figure S6: Western blot analysis of 4T1 cells incubated with different treatment groups; Figure S7: Images of 3D spheroids after 12 h of incubation with gelonin-488 nanoparticles; Figure S8: Quantitative analysis of 4T1 spheroid growth inhibition via CellTiter Glo assay (n = 3).
