# Supplementary material for: Intracellular Delivery of Therapeutic Protein via Ultrathin Layered Double Hydroxide Nanosheets
Source: Pharmaceutics. 2024 Mar 19;16(3):422. doi: 10.3390/pharmaceutics16030422 (PMC10974414; doi:10.3390/pharmaceutics16030422)
Supplement: Supplementary file 1 [file pharmaceutics-16-00422-s001.zip › pharmaceutics-2812671-supplementary.pdf]

Supporting Information

Intracellular Delivery of Therapeutic Protein Via Ultrathin Layered Double Hydroxide Nanosheets

He Zhang, Anle Ge, Yulin Wang, Boran Xia, Xichu Wang, Zhonghui Zheng, Changsheng Wei, Bo Ma, Lin Zhu, Rose Amal, Sung Lai Jimmy Yun and Zi Gu

Table S1 Results of gelonin loading on the LDH nanosheet.

Feed LDH (μg)	Feed gelonin (μg)	Residual gelonin (μg)	Loaded gelonin (μg)	Loading capacity (%)	Loading efficiency (%)
40	20	36.12683869	48.8731613	122.2	57.5

Table S2 Summary of hydrodynamic size, PDI, zeta potential and loading capacity of LDH and LDH-protein hybrids.

	Hydrodynamic size and PDI	Zeta potential	Protein loading capacity
LDH nanosheets	33 nm; 0.245	+26.7 mV	-
LDH-BSA	-	- 8.5 mV	182%
LDH-gelonin	44 nm; 0.19	+6.5 mV	122%

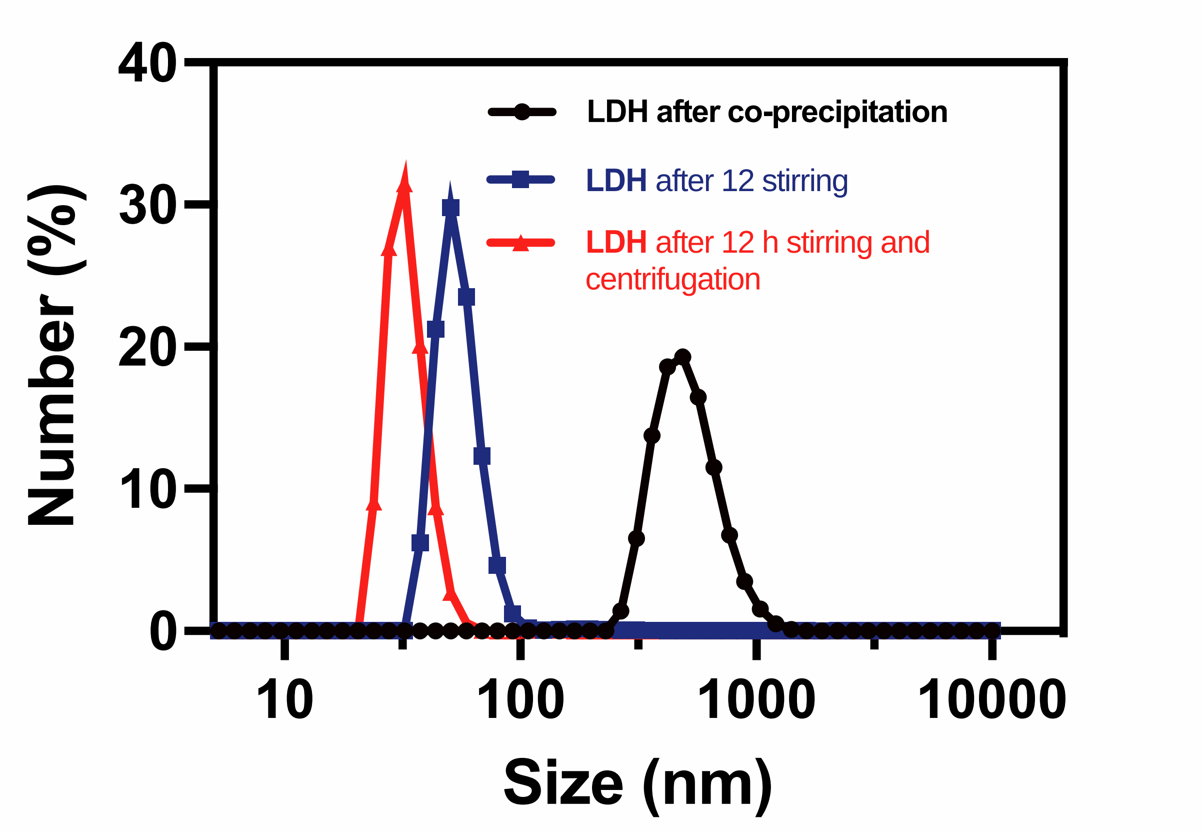

Figure S1 The size distribution of different samples.

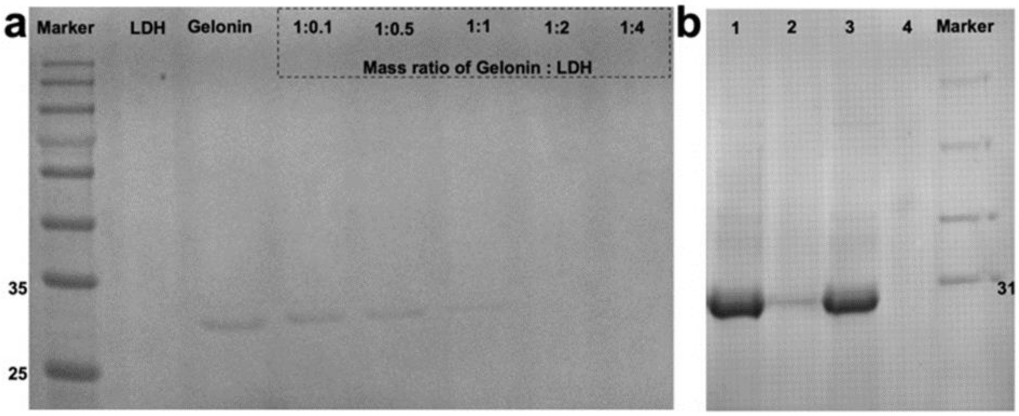

Figure S2 SDS-PAGE gels showing binding affinity and release study of LDH-gelonin. (a) The binding affinity of gelonin and LDH nanosheet with various mass ratios of gelonin to LDH nanosheets. (b) Evaluation of gelonin release capability from LDH nanosheets. Lane 1: gelonin (2 μg/μl) at pH 6.0; Lane 2: LDH-gelonin (1:1) at pH 7.4; Lane 3: LDH-gelonin (1:1) at pH 6.0; Lane 4: LDH nanosheets at pH 6.0.

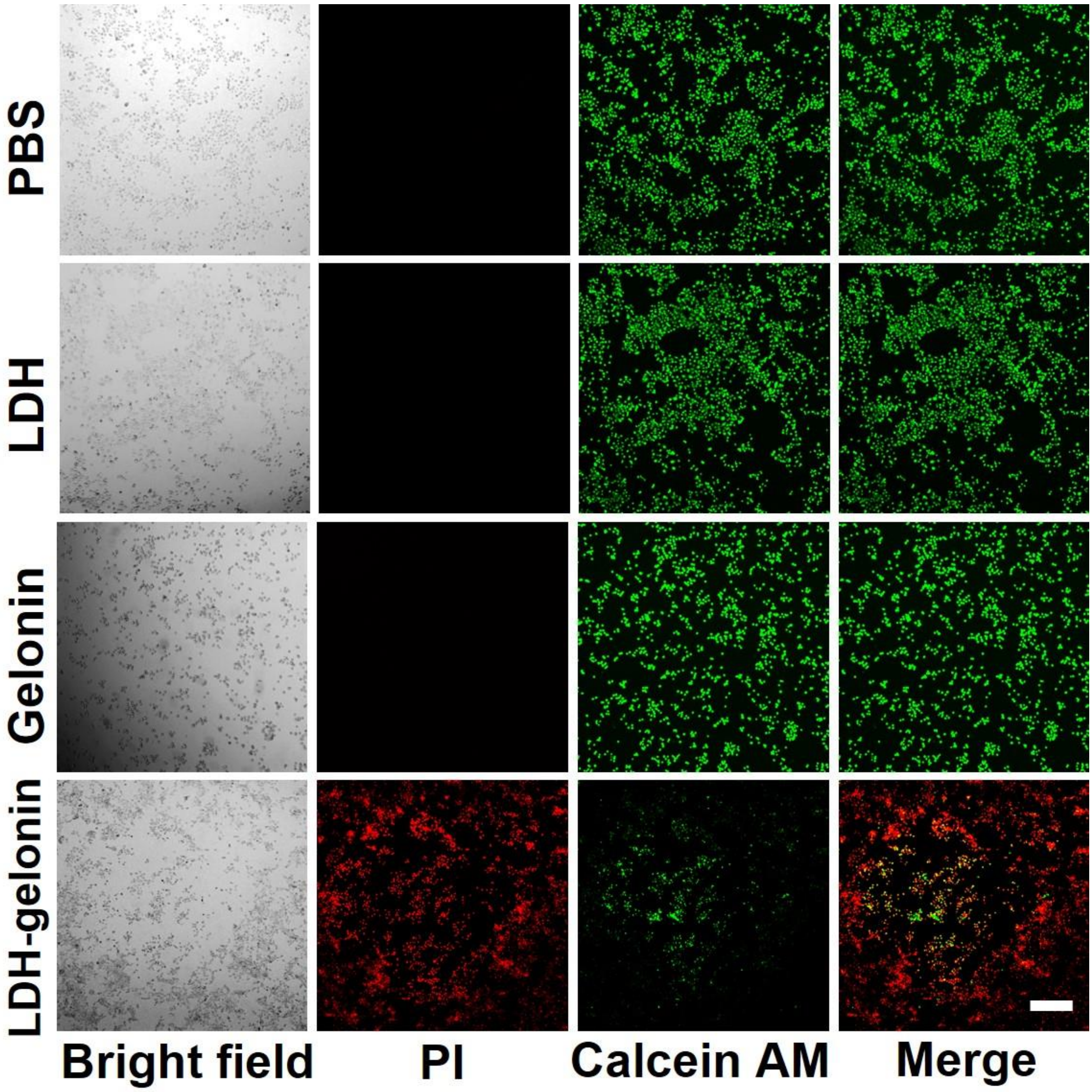

Figure S3. Fluorescence images of live and dead cells for 24 h incubation with PBS, LDH (5 μg/ml), gelonin (6 μg/ml), and LDH-gelonin (11 μg/ml). Green and red fluorescence indicate live and dead cells via Calcein AM and PI staining.

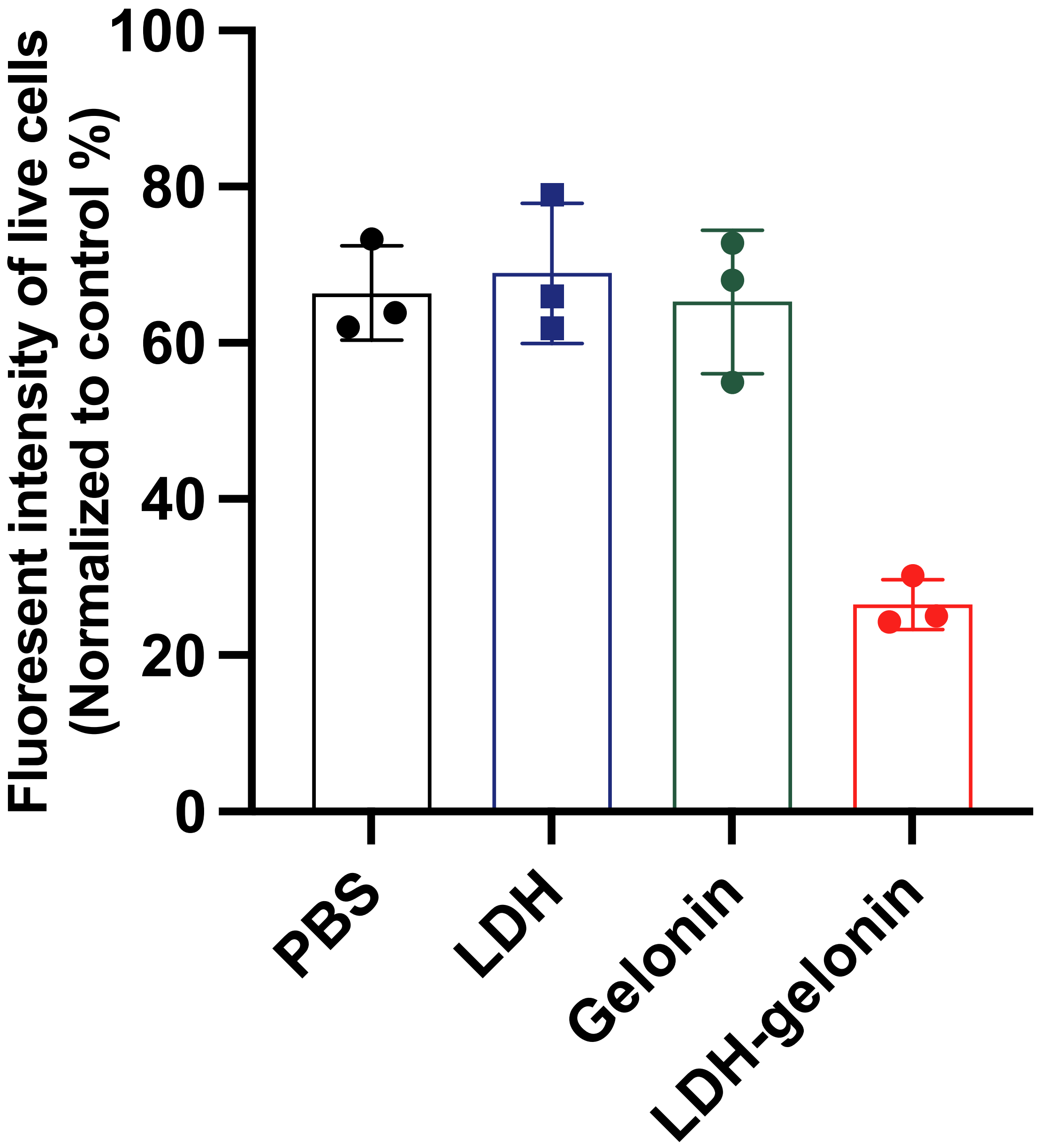

Figure S4 Quantification analysis of fluorescence image of live 4T1 cells incubated with PBS, LDH, gelonin or LDH-gelonin. Data was normalized to control group (n=3).

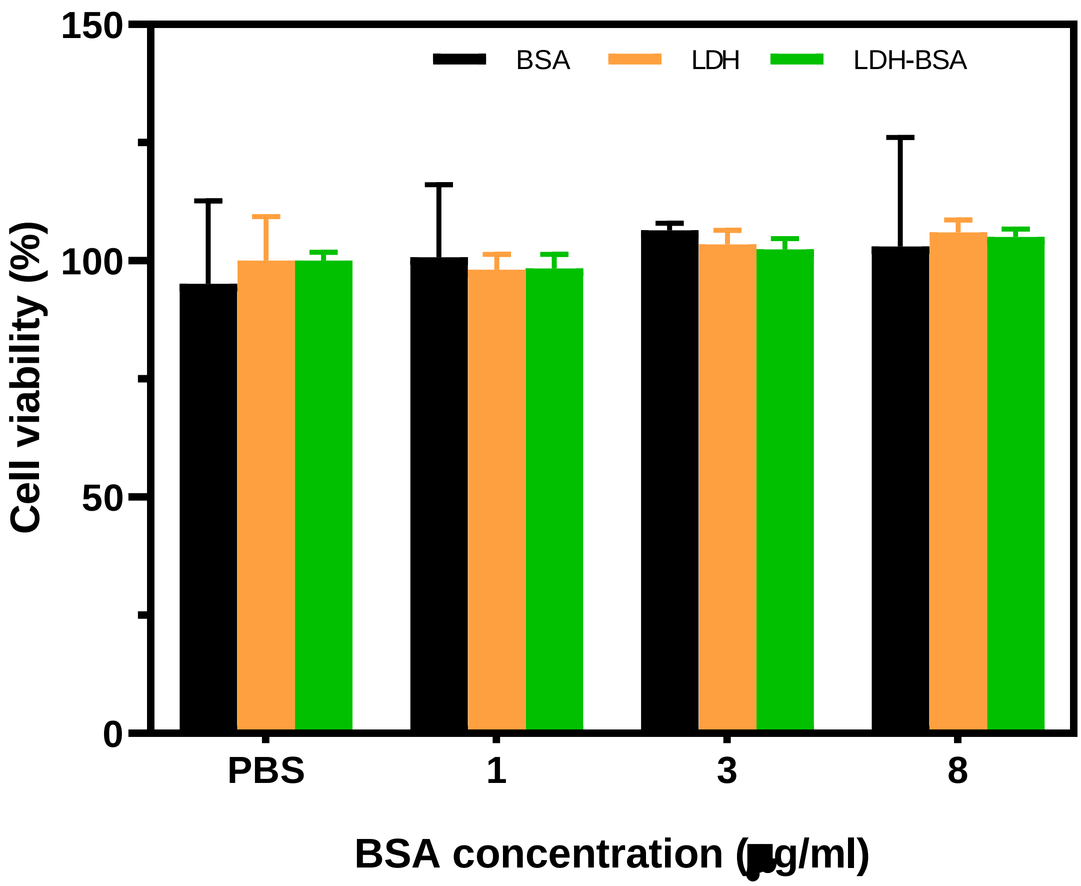

Figure S5 Cell viability of 4T1 incubated with PBS, BSA, LDH, and LDH-BDA with equivalent BSA concentrations (1-8 μg/ml) for 24 h (n = 3).

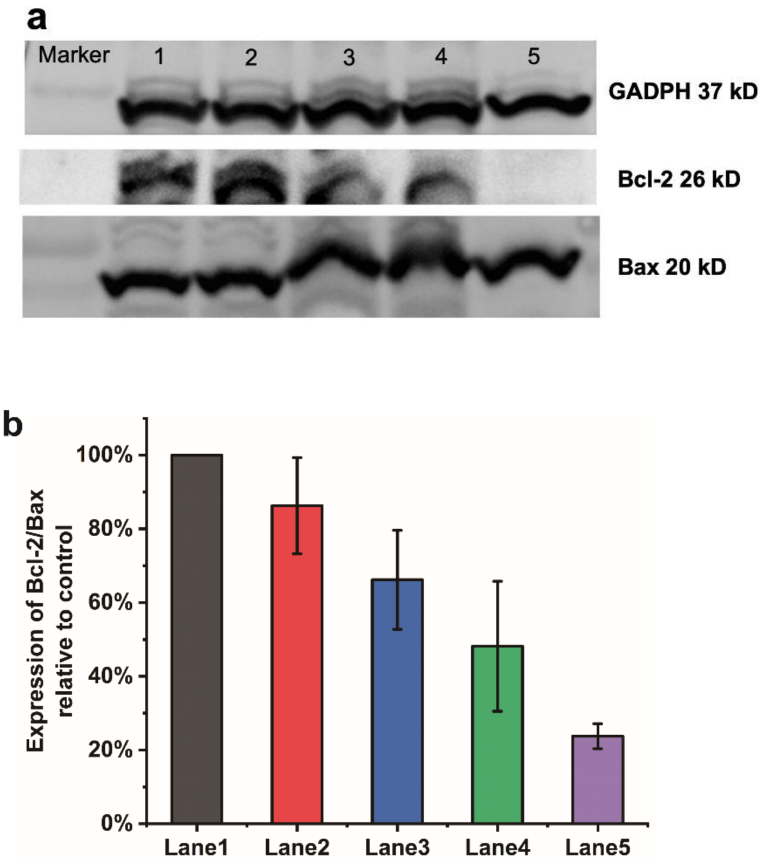

Figure S6 Western blot analysis of 4T1 cells incubated with different treatment groups. (a) Bcl-2 and Bax expression was examined by Western blot. GADPH expression was also examined to control for loading differences. Lane 1: Control group (PBS). Lane 2: LDH nanosheet (5 μg/ml). Lane 3: Gelonin (6 μg/ml). Lane 4: LDH-gelonin (5.5 μg/ml). Lane 5: LDH-gelonin (11 μg/ml). (b) Densitometric analysis of Bcl-2 protein relative to Bax protein. Data are presented as expression relative to control group (100%). Data were shown as the mean ± SD (n = 3).

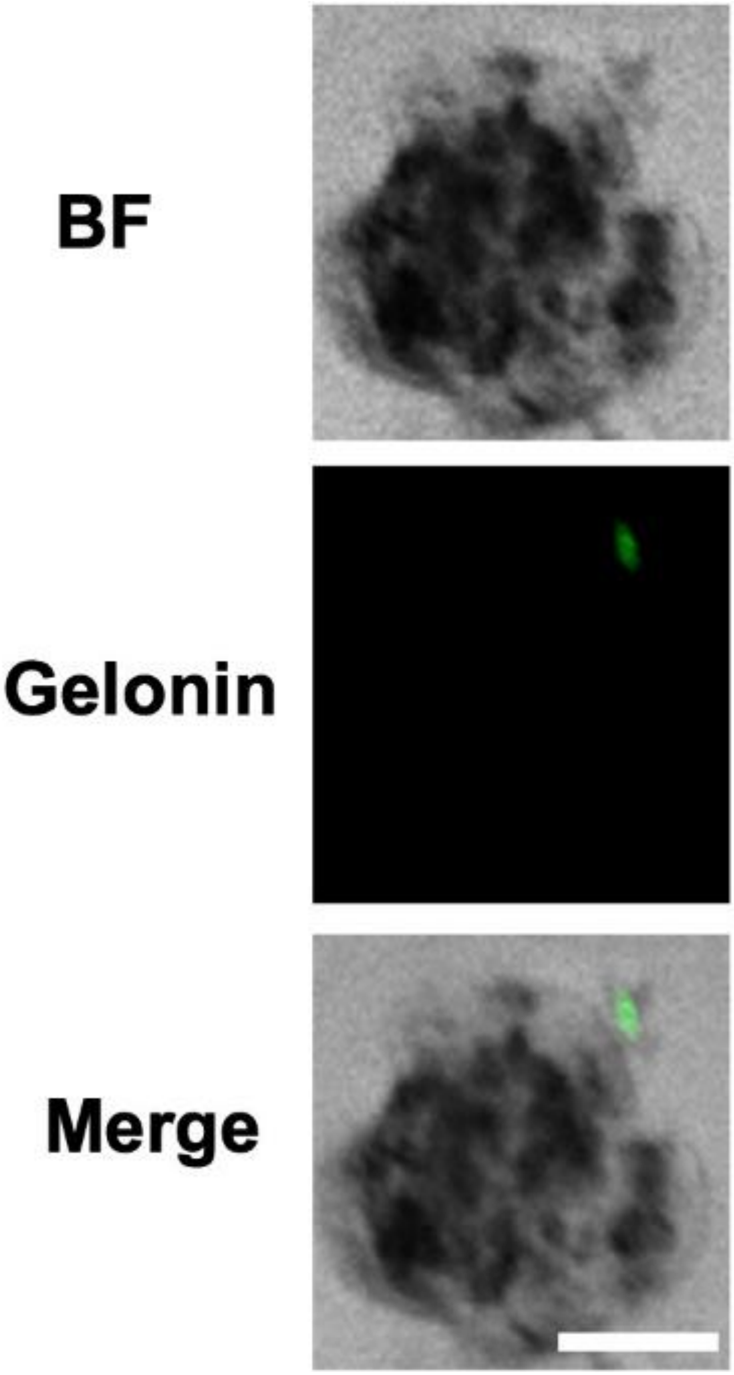

Figure S7 Images of 3D spheroids after 12 h of incubation with gelonin-488 nanoparticles. Scale bar = 50 μm.

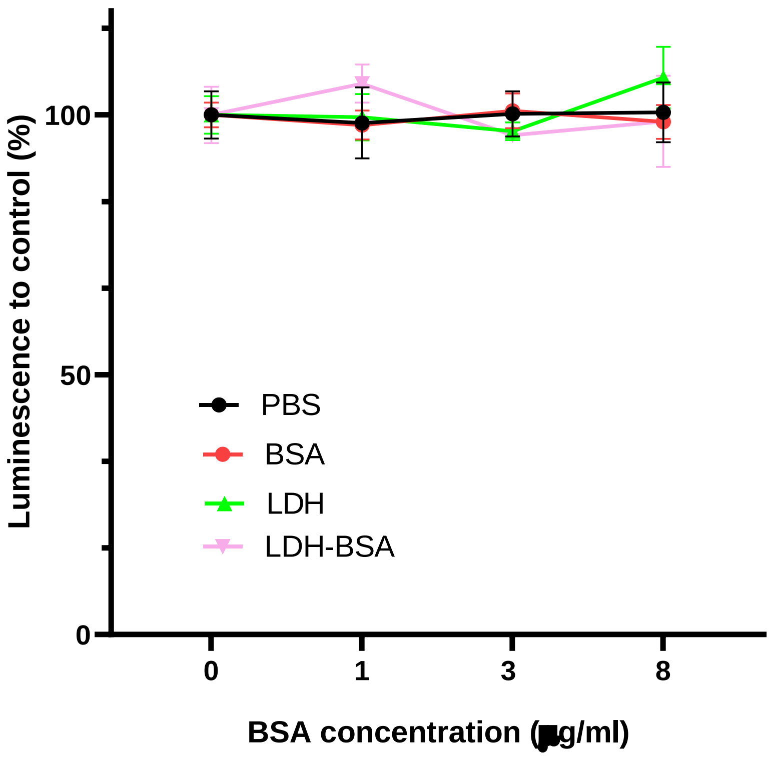

Figure S8 Quantitative analysis of 4T1 spheroid growth inhibition via CellTiter Glo assay (n=3).
